# Revisiting the humidity correction – A challenge to the current consensus

**DOI:** 10.1002/mp.70697

**Published:** 2026-10-06

**Authors:** Yuichiro Morishita, Hideki Yamaoka

**Affiliations:** ^1^ National Metrology Institute of Japan, AIST Tsukuba Ibaraki Japan; ^2^ TOYO MEDIC Co., Ltd. Nishi‐ku Osaka Japan

**Keywords:** air kerma, humidity correction, ionization chamber, radiation dosimetry, *W*‐value

## Abstract

**Background:**

The humidity correction factor, $k_{h}$, for ionization chambers in radiation dosimetry has relied on a consensus value established in ICRU Report 31 nearly half a century ago.

**Purpose:**

This study aims to rigorously re‐evaluate this long‐standing consensus by employing modern instrumentation.

**Methods:**

First, the equilibration time for the cavity air was examined by implanting a miniaturized humidity sensor directly into the cavity of a broken commercial ionization chamber to address uncertainties regarding stabilization in recent reports. Second, the $k_{h}$ was measured using a vacuum‐assisted gas exchange method, in which the air inside the cavity was exchanged actively. Finally, Monte Carlo simulations were performed to analyze energy deposition within the cavity, enabling the conversion of the measured $k_{h}$ to the relative W‐value.

**Results:**

The direct monitoring of humidity inside the cavity revealed that the equilibration time scale extended to several days, which was much longer than the time scales assumed in the recent reports. The measured $k_{h}$ for the Co‐60 beam exhibited slight but apparent differences from the long‐standing consensus: the measured $k_{h}$ extrapolated to 0 % relative humidity (RH) was 0.9962–0.9970, and the sudden response increase started from 0 % RH to 40 % RH (or possibly 30 % RH). The derived relative W‐value showed a roughly linear dependence on RH, which contrasted with the non‐linear behavior of the current consensus curve.

**Conclusions:**

Measurements using modern instrumentation indicate that the humidity dependence of $k_{h}$ and the corresponding relative W‐value for Co‐60 beam quality differ slightly from the long‐standing consensus established in ICRU Report 31. Although these differences are of minor importance for practical dosimetry, they may be relevant to primary air‐kerma standards. The present results suggest that primary standard laboratories may re‐evaluate their uncertainty budgets in light of these findings.

## 1 INTRODUCTION

Radiation dosimetry using air‐vented ionization chambers relies on the precise conversion of the measured ionization charge into SI‐traceable physical quantities such as air kerma^1^ in radiation protection dosimetry or absorbed dose to water^2^ in clinical dosimetry. This conversion requires corrections for environmental conditions, particularly temperature, pressure and humidity. While air density corrections based on temperature and pressure ($k_{\mathrm{TP}}$) are routinely applied and well‐standardized, the humidity correction, represented by $k_{h}$, is often treated as a static constant in an ordinary environment. However, since the physical definition of air kerma is strictly based on dry air, $k_{h}$ is not merely a minor correction but a fundamental component required to bridge the gap between the reference definition and measurements performed in ambient environments.

Despite its critical importance, the values of $k_{h}$ currently adopted by national metrology institutes (NMIs) and the Bureau International des Poids et Mesures (BIPM) have remained virtually unchanged for decades. Table 1 shows representative examples of $k_{h}$ values currently employed by several NMIs and BIPM in their Co‐60 air‐kerma standards.^3^ These institutes report nearly identical $k_{h}$ values with remarkably small uncertainties (typically ∼0.03 %). These values are primarily based on a series of experimental studies from the 1970s, specifically those by Niatel,^4^, ^5^ Guiho et al.,^6^ Referowski,^7^ and Hofmeester and Somerwil.^8^ As these foundational works, some of which were reported as internal CCEMRI documents, formed the basis for the consensus recommendation in ICRU Report 31,^9^ the resulting $k_{h}$ curve (hereafter referred to as ICRU31 curve) has been adopted by most metrology institutes as a practical standard. However, fully verifying the original experimental method and data from these decades‐old studies is increasingly difficult. This lack of accessible detail makes it challenging to re‐evaluate $k_{h}$ based on contemporary metrological requirements.

**TABLE 1:** Humidity corrections ($k_{h}$) and their standard uncertainties ($\delta k_{h}$) adopted by metrology institutes in several countries for air‐kerma standards with Co‐60 gamma rays. Relative standard uncertainty of air kerma ($\delta K_{a}$) and published year are also tabulated.

Country	Institute*	$k_{h}\left(\delta k_{h}\right)$	$\delta K_{a}$ (%)	Year
——	BIPM	0.9970(3)	0.13	2025
AUS	ARPANSA	0.9971(4)	0.21	2021
BRA	LNMRI/IRD	0.9970(3)	0.39	2005
CAN	NRC	0.9970(5)	0.19	2022
DEU	PTB	0.9970(3)	0.19	2014
FRA	LNE‐LNHB	0.9970(3)	0.32	2013
GBR	NPL	0.9970(5)	0.28	2019
JPN	NMIJ	1.0000(2)	0.23	2022
KOR	KRISS	0.9970(3)	0.18	2022
RUS	VNIIM	0.9970(3)	0.19	2011
USA	NIST	0.9971(6)	0.30	2025

* The full names of the institutes are available on the BIPM website.^3^

Recently several research groups have reported experimental results regarding $k_{h}$ that conflict with the long‐standing consensus. Poirier and Douysset^10^ report that the response of a well‐type ionization chamber (Nucletron 077.091) unexpectedly increases by approximately 0.7 % as relative humidity (RH) rises from 20 % to 90 % for an Ir‐192 source. This observation contradicts the ICRU31 curve both in terms of magnitude and the overall trend of the response. McEwen and Taank^11^ report that several reference ionization chambers (PTW TN‐30013, IBA FC65‐G and FC65‐P, and Exradin A19) do not exhibit the response change as low as 5.5 % RH (mixing ratio 2.5 g/kg at 40 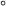 C) for Co‐60 and Sr‐90 sources. Similarly, Sato et al.^12^ report that the PTW TN‐30013 and Exradin A19 ionization chambers do not show the response change as low as 10 % RH under Linac beam qualities. Although the experimental uncertainties in the latter case might partially mask the expected humidity effect at 10 % RH, the broader humidity range down to 5.5 % RH investigated in the former study is sufficient to distinguish the $k_{h}$ effect. However, their findings are not entirely definitive; although no apparent change is observed to support the ICRU31 curve, the data exhibit subtle trends that complicate a simple interpretation. The discrepancies between the pioneering and the recent reports raise a fundamental question: Why is the humidity correction prescribed by the ICRU31 curve not consistently observed in contemporary experiments despite the use of sophisticated modern instrumentation?

To resolve the discrepancies and derive a plausible $k_{h}$ correction using the modern instrumentation in this study, we first addressed a question raised in the recent reports and subsequently measured the ionization current as a function of the RH in the ionization cavity.

## 2 METHODS

### 2.1 Test bench for measurements

A climatic cabinet of 50 cm cubic shape with 3‐mm‐thick polyvinyl chloride (PVC) walls, shown in Figure 1(a), was used as a test bench for measurements in this study. The cabinet was configured with two specialized panels on opposite sides. One side featured a hinged door for manual access directly inside the cabinet, while the opposite side served as a multi‐functional interface housing (1) signal and sensor feedthroughs, (2) feedthroughs for humidity‐conditioned air and temperature‐conditioned water, and (3) an integrated rubber glove, which allows for internal manipulation without compromising the sealed environment.

**FIGURE 1:**
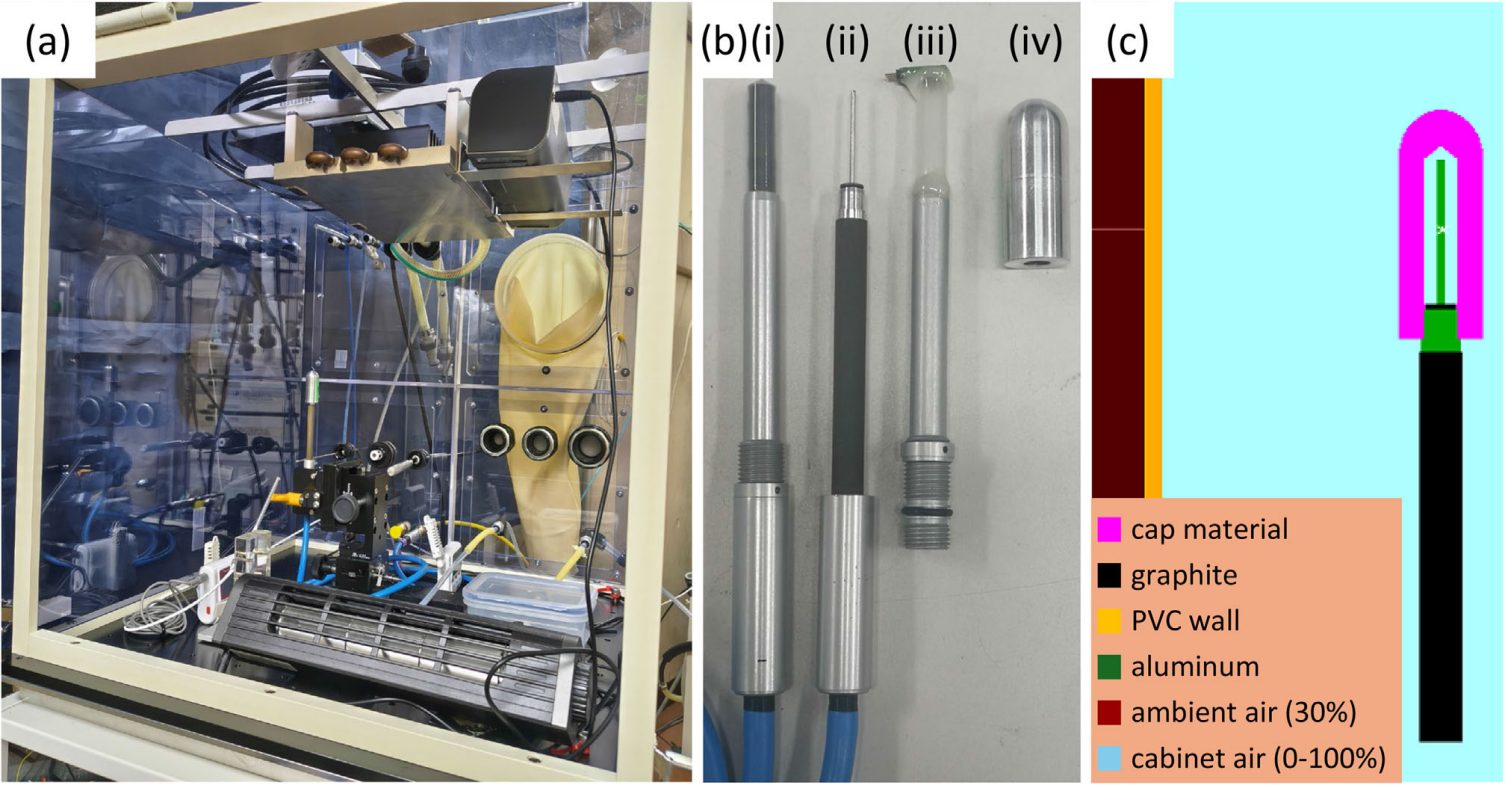
(a) Test bench for the measurements. The rubber glove and some feedthroughs for sensors, water, air and vacuum are located on the side panel. (b) Components of the ionization chamber: (b‐i) the original functional 30013 chamber (not used in this study), (b‐ii) the decommissioned 30013 chamber with the outer shell and cavity wall removed, (b‐iii) the outer shell and a substitute wall equipped with the small humidity sensor, and (b‐iv) the aluminum metal cap. (c) Cross‐sectional EGSnrc model of the cabinet and the ionization chamber. The materials assigned to each component are also indicated.

The RH and temperature inside the cabinet were regulated via independent proportional‐integral‐derivative (PID) controls. Ambient air was compressed to approximately 0.7 MPa by a compressor, resulting in dehydration. The resulting dry air was delivered to a humidity controller (AHCU‐2, Kitz Micro Filter), which adjusted the humidity and pressure to supply conditioned air to the cabinet. For temperature regulation, water from a thermostatic bath was circulated through a finned heat sink mounted on the ceiling of the cabinet. To enable independent feedback control, humidity and temperature sensors placed inside the cabinet were routed through the feedthroughs to their respective controllers. Finally, to ensure spatial uniformity of both temperature and humidity, two internal fans were installed on the ceiling and the floor of the cabinet to facilitate constant air circulation. The cabinet was designed to be airtight; however, one of the feedthroughs was equipped with a variable leak valve to allow for precise regulation by the humidity controller. This integrated system allowed for a wide humidity control range, from a few percent RH up to 80 % RH. Under these conditions, the internal environment was stabilized within ± 0.5 % for RH and 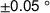 C for temperature throughout the measurements.

A small airtight box (12×9×5cm) was installed in the cabinet. The box was equipped with two gate valves: one connected to a vacuum pump through a dedicated feedthrough for evacuation, and the other opening into the inside of the cabinet to intake the humidity‐conditioned air. A brief 10‐second evacuation was sufficient to reduce the internal pressure from atmospheric pressure to approximately 6 kPa.

Inside the cabinet, a holder was positioned to fix the ionization chamber detailed below. The ionization chamber was located 5 cm from the cabinet wall. Temperature, pressure and humidity inside the cabinet were measured with a PTU‐303 by Vaisala with traceability to the primary standards of Japan. An additional Pt‐100 thermometer (in‐house calibrated) was then arranged on the opposite side of the holder relative to the PTU‐303 to verify the temperature gradient across the holder.

### 2.2 Measurements

Two distinct types of measurements were performed in this study. In both measurements, an ionization chamber TN‐30013 by PTW with a damaged outer cavity wall was utilized. This specific chamber model was selected not only because its response was shown to be stable against humidity changes in our previous study^13^ but also due to its long‐term operational stability over years of use in our laboratory.

#### 2.2.1 Equilibrium time measurement I

To verify the actual equilibrium time for the cavity air, we modified the damaged ionization chamber to enable direct internal monitoring. As shown in Figure 1(b‐iii), the original damaged wall was replaced with an equivalent substitute wall unit equipped with a compact humidity sensor, HS4001 by Renesas, (hereafter, components in Figure 1(b) are referred to by their labels alone, for example, (b‐i), (b‐ii), and so on). To characterize the humidity response of this newly introduced unit, we evaluated two configurations: (1) the substitute wall unit alone (b‐iii) and (2) the reassembled chamber (b‐ii+b‐iii), which is functionally equivalent to the original commercial chamber (b‐i). These assemblies were then placed inside the cabinet and exposed to cyclic humidity changes between ∼20 % RH and ∼80 % RH.

#### 2.2.2 Humidity correction measurement II

Based on the results obtained in measurement I, we concluded that passive equilibration through molecular diffusion was impractical for determining the humidity correction, $k_{h}$, for the original chamber (b‐i). Therefore, we employed an active equilibration method using vacuum‐assisted gas exchange described below. For this purpose, the aluminum metal cap (b‐iv) was utilized, which functions as both a build‐up cap and an external electrode. The selection of aluminum was primarily based on its mechanical durability. Since the vacuum‐assisted gas exchange process required frequent attachment and removal of the cap, the use of a fragile graphite cap was avoided, as it could not ensure a constant chamber response due to potential abrasion. The metal cap has a wall thickness of 4.5 mm, designed to provide sufficient electronic equilibrium for the Co‐60 beam quality used in this study.

The active gas exchange process was performed as follows: the metal cap alone was placed inside the small airtight box. The box was evacuated to below 6 kPa by the vacuum pump and then refilled with humidity‐conditioned air from the cabinet. This evacuation‐refilling cycle was repeated 5 times to ensure the complete replacement of the air within the cap. After the cycles, the metal cap was promptly attached to the ionization chamber body (b‐iv), which remained firmly mounted on the holder throughout the measurement II (as depicted in Figure 1(a)), by hand using the integrated rubber glove.

The irradiations were performed using the Co‐60 source at the National Metrology Institute of Japan (NMIJ) and the source‐to‐chamber distance was ∼1 m with a circular radiation field of 11 cm diameter. Measurements at each humidity level were conducted over a 24‐hour period. On the day before a measurement, the ambient humidity within the cabinet was set to the target value, and the metal cap was left detached overnight to ensure pre‐equilibration. On the following morning, a series of measurements consisting of seven consecutive cycles was performed for the specified humidity. Each cycle began with the vacuum‐assisted gas exchange and the attachment of the metal cap to the chamber body as described above. Subsequently, a bias voltage of 300 V was applied to the central electrode of the ionization chamber using an electrometer (520, EMF Japan). After a 10‐minute stabilization inside the cabinet, the ionization current was measured 10 times at 5‐minute intervals. Each individual measurement consisted of a pair of sequences: one during irradiation and one without irradiation. The latter was used to determine the leakage current, which was then subtracted from its corresponding total current measured during irradiation to obtain the net ionization current. Each sequence comprised 100 current readings at a 2 Hz sampling rate. During the measurement sequences, the temperature, pressure, and humidity were recorded at 1 Hz. Once the 10 measurements in a cycle were completed, the high voltage was turned off, and the procedure returned to the evacuation‐and‐refilling step for the next cycle. After the full seven cycles were completed, the system was transitioned to the next humidity level and left overnight. The environmental parameters inside the cabinet were recorded every minute throughout the measurement II.

### 2.3 Monte Carlo simulations

Monte Carlo (MC) simulations were performed to determine the energy deposition to the cavity air. These results were used to convert the measured ionization current into the relative W‐value of the air, enabling a direct comparison with the previous studies. The programs were developed using an egs++ sample code (egs_chamber) in the EGSnrc code system.^14^ The geometry of the ionization chamber was modeled based on direct measurements of the dimensions of each component shown in Figure 1(b‐ii). A cross‐sectional view of the resulting EGSnrc model of the ionization chamber together with the irradiation cabinet is shown in Figure 1(c), where the materials assigned to each component are also indicated. The material of each component was assigned based on its external appearance. Internal structures that could not be identified were not modeled; instead, each component was treated as a homogeneous body of the assigned material. The Co‐60 source was modeled as a point source positioned 1 m from the ionization chamber. The incident photon spectrum, including photons scattered from the collimator, was taken from a previous study.^15^ The beam was defined to produce a circular field with a diameter of 11 cm at the chamber position. Special attention was paid to the preparation of the material data for humid air with an RH of h [%]. The Wagner and Pruss formula^16^ was used to determine the partial pressures of vapor $p_{v}\left(h\right)$ [Pa] and dry air $p_{\mathrm{dry}}\left(h\right)=101325-p_{v}\left(h\right)$ (see Appendix for details). The dry air was a mixture of nitrogen, oxygen, argon, carbon dioxide, neon and others with a mixing percentage rate of 78.084, 20.9476, 0.934, 0.0314, 0.00182 and 0.00118, respectively.^17^ Since the former five gases account for 99.99882 % of the total composition, we assumed these gases as the dry air for the input of the MC simulations with the renormalized mixing rate, which was also applied to humid air with $p_{\mathrm{dry}}\left(h\right)$ to calculate the partial gas pressures of $p_{x}\left(h\right)$ ($x=N_{2},O_{2}$, etc). The masses of the gases were calculated using the ideal gas formula given by $p_{x}m_{x}/RT$, where $m_{x}$, R and T denote the molar mass, the ideal gas constant and the gas temperature, respectively. The reference data by Commission on Isotopic Abundances and Atomic Weights was used to calculate the molar masses.^18^ The gas density and the mass fractions required for the MC input were calculated from these masses. The density correction, which is also required for the MC input, was evaluated using a Fortran program, ESTAR version 1.2, distributed by National Institute of Standards and Technology (NIST).^19^ The mass fraction and density calculated above were used for the input of the program, and we used the default mean excitation energy as predicted by the program. It was found that the density correction calculated had a very weak dependence on humidity ranging from 0 % RH to 100 % RH and was zero for electron and positron kinetic energies below 30 MeV. Accordingly, the density correction for humid air was not incorporated in the MC simulations. The default transport parameters and simulation options provided by EGSnrc were used in the MC simulations.

The simulations were performed by varying the RH in both the cabinet and the chamber cavity from 0 % to 100 % RH, while maintaining the RH outside the cabinet at 30 % RH. The energy deposition to the cavity air was then scored. In addition to the energy‐deposition simulations, further MC simulations were conducted to evaluate the influence of the cap material. The aluminum cap was replaced with graphite and yellow brass (Cu: 66 wt% and Zn: 34 wt%), and the volumetric electron spectra in the chamber cavity were scored for each configuration.

### Declaration of Generative AI

During the preparation of this manuscript, the authors used Gemini (Google) to improve the English grammar, readability of the text and the logical flow. After using the AI tool, the authors reviewed and edited the content as needed and take full responsibility for the content of the published article.

## 3 RESULTS

### 3.1 Equilibrium time measurement I

The solid line in Figure 2(a) shows humidity response within the cavity of the outer shell and wall unit (b‐iii) in Figure 1 when the humidity in the cabinet (dashed line) was cyclically varied between 17 % RH and 80 % RH. Although the humidity in the cabinet reached the target settings within 2 hours, the response in the cavity was significantly slower. Notably, the rates of the humidity changes in the cavity appeared to be consistent between the increasing and decreasing phases, suggesting a similar diffusion mechanism regardless of the direction of the humidity change.

**FIGURE 2:**
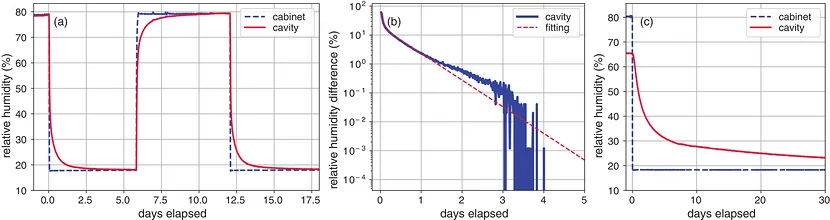
Measured humidity responses within the cavity of the ionization chamber. (a) Cyclic humidity response of the outer shell and wall unit (Bc). The solid and dashed lines represent the humidity in the cavity and the ambient humidity in the cabinet, respectively. (b) Semi‐logarithmic plot of the humidity change from the final equilibration value for the (b‐iii) unit. The solid and dashed lines represent the experimental data and the fitting curve, respectively. (c) Long‐term humidity response of the assembled unit (b‐ii)+(b‐iii).

To visualize the equilibration process in detail, humidity change in the cavity during the decreasing phase was re‐plotted on a semi‐logarithmic scale. The solid line in Figure 2(b) shows the time evolution of the humidity difference from the final equilibrium value. The plot clearly demonstrates that the humidity change is governed by multiple time constants. The data were fitted with a double‐exponential function with two time constants and the resulting fit is shown by the dashed line in Figure 2(b). The obtained time constants were $T_{1}=0.038$ d and $T_{2}=0.47$ d. It is important to note that $T_{2}$ is more than 10 times longer than $T_{1}$, which delays the overall equilibration.

The solid line in Figure 2(c) shows a plot similar to Figure 2(a) for the assembled unit (b‐ii+b‐iii). Although the initial humidity in the cavity had not reached equilibrium with the ambient humidity shown by the dashed line in Figure 2(c), the measurement was started. This was due to practical reasons, including time constraints and the operational difficulties for fully equilibrating the cavity humidity with the surroundings. In this measurement, the humidity was monitored for a long‐term period of 30 days after the humidity in the cabinet was changed from 80 % RH to 17 % RH. It was observed that it took approximately one week for the RH difference between the cavity and ambient air to reach 10 % RH. By this time, the rate of humidity change had gradually decreased to less than 1 % RH/d, indicating that reaching full equilibrium would require a much longer duration.

### 3.2 Monte Carlo simulations

The closed circles in Figure 3(a) show the relative energy depositions to the cavity air calculated by the MC simulations and plotted as a function of the RH in both the cabinet and the chamber cavity from 0 % to 100 % RH. The RH outside the cabinet was fixed at 30 % RH throughout the simulations. The error bars indicate the statistical uncertainties (k=1). For comparison, the closed squares represent the relative air density in the chamber cavity.

**FIGURE 3:**
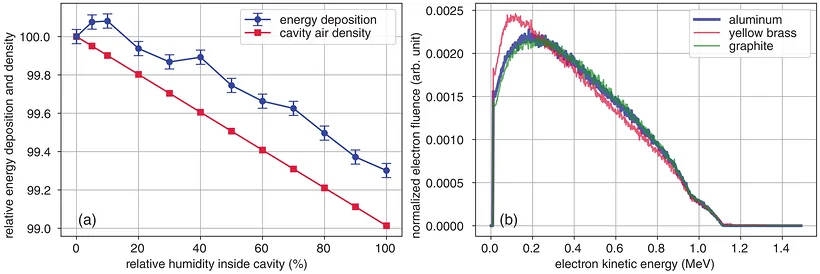
(a) Simulated relative energy deposition to the cavity air (•) and relative air density (▪) as a function of the RH in the chamber cavity. (b) Normalized volumetric electron spectra inside the cavity for different cap materials of the same thickness, normalized to their respective total fluence to compare the spectral shapes.

Figure 3(a) reveals one notable feature: the relative energy depositions are roughly proportional to the relative air density. This trend arises because most secondary electrons generated in the cavity wall possess sufficient energy to penetrate the air in the chamber cavity. However, a fraction of lower‐energy electrons deposit their entire energy within the cavity air regardless of the RH, leading to the observed deviation from strict proportionality to the air density.

Figure 3(b) compares the normalized volumetric electron spectra inside the cavity for different cap materials (aluminum, graphite and yellow brass) of the same thickness. The RH in both the cabinet and the chamber cavity was fixed at 0 % RH. Although a slight difference is observed, particularly for yellow brass in the low‐energy region, the overall spectral distributions are in good agreement irrespective of the cap material.

### 3.3 Humidity correction measurement II

#### (i) Ambient conditions and leakage current

Figure 4 shows a representative 1.5‐day data set recorded during measurement II, exhibiting the temporal stability and the system's response to the manual interventions during the vacuum‐assisted gas exchange process. Figures 4(a)–(c) display the ambient temperature, pressure and RH within the cabinet, respectively. The horizontal axis represents the elapsed days with the time origin ($t_{0}=0$) defined as 00:00:00 on the first day of the response measurements described below. This reference time also serves as the basis for the radioactive decay correction of the Co‐60 source applied in the subsequent analysis. The temperatures were monitored at two different positions to verify the spatial temperature gradients in the cabinet. The temperature difference between the sensors remained within 0.1 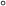 C throughout the measurement. The temperature was maintained at 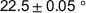 C except for periodic spikes. The periodic spikes observed in Figures 4(a)–(c) correspond to the vacuum‐assisted gas exchange processes. During these procedures, the operator's arm and hand were inserted into the cabinet via the integrated rubber glove to handle the aluminum metal cap (Bd). Each manual intervention caused a slight temperature rise of 0.2 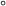 C due to body heat, which in turn led to a corresponding decrease in RH. The magnitude of this RH decrease depended on the RH level: it was negligible below 10 % RH, approximately 0.2 % RH at 20 % RH, and increased to nearly 1 % RH at 80 % RH. Additionally, the humidity‐conditioned air supply could not keep pace with the volumetric displacement of the arm, leading to momentary pressure fluctuations.

**FIGURE 4:**
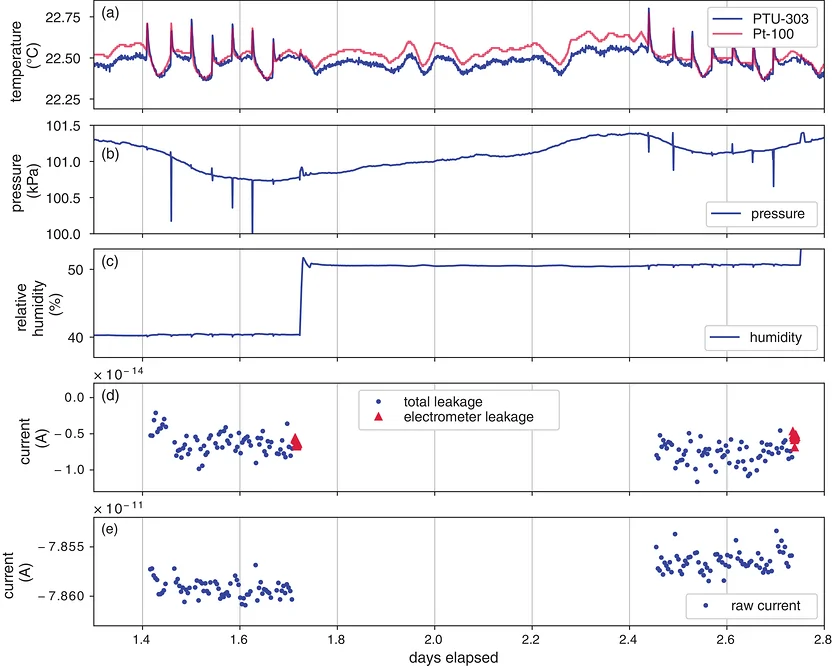
Representative 1.5‐day data set during the measurement II: (a) ambient temperatures, (b) pressure, (c) RH, (d) leakage currents (•: total system, ▴: electrometer), and (e) raw signal currents during irradiation.

The closed circles in Figure 4(d) show the averaged leakage currents measured in pair with the signal currents in Figure 4(e). Following the vacuum‐gas exchange process and a 10‐minute stabilization, measurements were performed 10 times at 5‐minute intervals. The closed triangles in Figure 4(d) show the leakage currents of the electrometer alone, measured by disconnecting the ionization chamber from the electrometer.

Figure 5 shows overall leakage currents of the total system (•) and those of the electrometer itself (▴) as a function of RH. The total leakage currents remained stable at approximately $-7\times 10^{-15}$ A, showing no sensitivity to the RH. Notably, the leakage of the total system was consistent with that of the electrometer itself, indicating that the contribution of the ionization chamber to the total leakage was negligible despite the common expectation that increasing cavity humidity would increase the leakage current.

**FIGURE 5:**
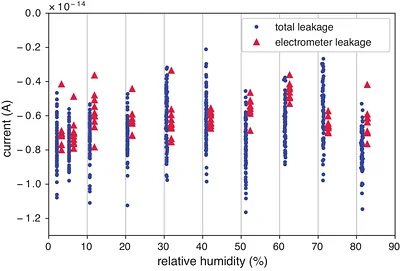
Leakage currents of the total system (•) and the electrometer itself (▴: offset horizontally for clarity) as a function of RH.

#### (ii) Normalized current and uncertainty evaluation

Figure 4(e) shows the averaged raw currents measured during irradiation. The very slight decrease in the magnitude of the current on the second day compared to the first day is due to the Co‐60 decay. Although the metal cap was removed from and reattached to the ionization chamber for each vacuum‐gas exchange process, no apparent deviation was observed relative to the inherent fluctuations of the signal itself within each measurement cycle.

To evaluate the humidity dependence of the response, $k_{h}$, the raw signal current, $I_{R}$, was corrected for the paired leakage current, $I_{L}$, the air density and the radioactive decay. The normalized ionization current is defined as

(1) $$I_{\mathrm{norm}}=\frac{\left(\frac{T}{295.15}\right)\left(\frac{101.325}{P}\right)\left(I_{R}-I_{L}\right)}{{\left(1/2\right)}^{\left(t-t_{0}\right)/T_{1/2}}},$$

where P and T are the pressure in kPa and temperature in K, t is the time at each measurement, $t_{0}$ is the reference time, and $T_{1/2}$ is the half‐life of the Co‐60 (5.2711×365.2422 d).^20^

The uncertainty of $I_{\mathrm{norm}}$ was evaluated by considering both Type A and Type B components, which are tabulated in Table 2. For the Type B uncertainty in T, the intrinsic calibration uncertainty (0.03 K) and the temperature gradients (0.05/$2\sqrt{3}$ K) were taken into account. Since no calibration data were available in the range of $I_{L}$, the Type B uncertainty in $I_{L}$ was adopted as $3\times 10^{-17}$ A based on the calibration performance at $1\times 10^{-11}$ A (the lowest calibrated current level available). The beam output from the therapy‐level Co‐60 source was sufficiently stable during the measurements. Since no parameters related to the source position or irradiation geometry were changed throughout the experiment, beam stability was evaluated by considering only the change in photon attenuation in ambient air. Although air attenuation depends on both atmospheric pressure and temperature through the air density, the ambient temperature was controlled within ±0.1 K throughout the measurements, and its contribution was negligible compared with that of the atmospheric pressure. The recorded atmospheric pressure varied between 100 kPa and 102 kPa, and the corresponding Type B uncertainty for beam stability was evaluated using the mass attenuation coefficient^21^ of air for the mean Co‐60 photon energy (1.25 MeV), assuming a rectangular distribution. The resulting uncertainty is listed as “beam stability” in Table 2. Except for those described above, all Type B uncertainties were obtained from calibrations by metrological institutions or the evaluations in the literature.^20^ The uncertainty propagation was calculated rigorously for each individual RH point using the automatic differentiation via the JAX library.^22^ The resulting overall relative uncertainties were approximately 0.02 % (k=1).

**TABLE 2:** Uncertainty budget of the normalized current (k=1).

Quantity	Type	Value	Uncertainty	Unit
$I_{R},I_{L},T,P$	A	$-7.8\times 10^{-11}$	$6\times 10^{-15}$	A
$I_{R}$	B	$-7.8\times 10^{-11}$	$9\times 10^{-14}$	A
$I_{L}$	B	$-8\times 10^{-15}$	$3\times 10^{-17}$	A
P	B	101.3	0.01	kPa
T	B	295.7	0.033	K
$T_{1/2}$	B	1925.23	0.29	d
Beam stability	B	1.00000	0.00004	—
Total		$-7.8\times 10^{-11}$	$1.5\times 10^{-14}$	A

#### (iii) Humidity correction factor and relative *W*‐value

Based on the normalized currents and their uncertainties, the relative response, $k_{h}$, of the ionization chamber was defined as the ratio of $I_{\mathrm{norm}}$ to its average value at 40 % RH. This relative response serves as the experimental $k_{h}$ value in the subsequent analysis. The small dots with error bars, labeled as “individual data” in Figure 6(a), represent this relative response measured for each measurement cycle as a function of the internal RH of the cavity. These points are normalized to the average value at 40 % RH and are horizontally offset by 1 % RH for visual clarity. The error bars for these individual points account for only the Type A uncertainties. By averaging the data points at each RH level and incorporating the Type B uncertainty components, the closed circles with error bars, labeled as “all data” in Figure 6(a), show the conclusive $k_{h}$ correction factor. The typical overall uncertainty for these points is approximately 0.02 % (k=1).

**FIGURE 6:**
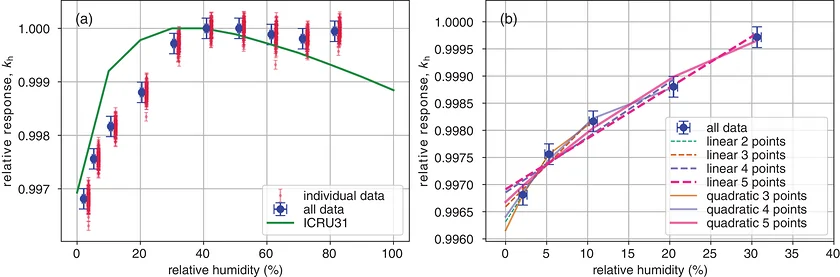
(a) Relative response, $k_{h}$, of the ionization chamber as a function of the internal RH within the cavity. The small dots (horizontally offset for clarity) and closed circles represent the individual and averaged data, respectively. The solid line shows the ICRU31 curve for comparison. (b) Expanded view of the low‐humidity region below 40 % RH. The dashed and solid lines represent the linear and quadratic fits, respectively, using a variable number of data points (n=2−5) starting from the lowest humidity.

Figure 6(b) shows $k_{h}$ with a focus on the low‐humidity range below 40 % RH to investigate the extrapolation to 0 % RH required for the definition of the SI‐unit of air kerma. In this panel, both linear (dashed lines) and quadratic (solid lines) fits were performed by varying the number of data points, n=2−5, starting from the lowest humidity. To facilitate visual comparison, the fits using the same n are plotted with the same color and line thickness. The intercepts of the fits at 0 % RH range from 0.9962 to 0.9970.

Subsequently the relative W‐value, defined as the average energy required to create an ion pair, was evaluated as the ratio of the energy deposition shown in Figure 3(a) to the experimental $k_{h}$ shown in Figure 6(a). For each humidity value in Figure 6(a), the corresponding energy deposition was determined by interpolation of the results in Figure 3(a). The closed circles in Figure 7 show the evaluated relative W‐values (vertically offset for comparison) as a function of RH. In addition, the experimental and theoretical results reported by Niatel^4^, ^5^ and by Barnard et al.^23^ are also plotted as closed triangles and squares, respectively. These literature data were digitized from ICRU Report 31^9^ and converted to the RH using formulas provided in the Appendix.

**FIGURE 7:**
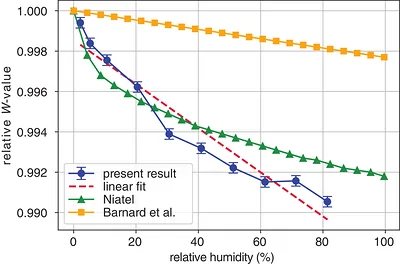
Relative W‐value as a function of RH. The closed circles (vertically offset for comparison) were obtained from the ratio of the energy deposition by the MC simulation to the measured $k_{h}$ value. The closed triangles and squares show the experimental and theoretical results by Niatel and Barnard et al. respectively.

## 4 DISCUSSION

### 4.1 Equilibrium time measurement I

To interpret the results in Figure 2, the following points should be kept in mind: (1) because the air in the cabinet was circulated by the fans or natural convection induced by temperature gradients, equilibration of the air inside the cavity progressed not only through molecular diffusion but also through the direct exchange of air facilitated by the air flow. (2) the material used for the cavity wall (polymethyl methacrylate) is capable of transmitting or absorbing water molecules,^13^ which may introduce additional time‐dependent responses. (3) the structural differences significantly affect the air exchange process; the dead‐end cavity is located at the end of a long (∼1.5 m) and narrow path in the assembled unit (b‐ii+b‐iii), whereas it is accessed via a much shorter and wider path in the outer shell and wall unit (b‐iii).

Based on the considerations above, the multi‐exponential behavior observed for the (b‐iii) unit in Figure 2(b) can be interpreted as follows. Given that the (b‐iii) unit has a relatively short and wide opening, the air near the entrance is quickly replaced by the humidity‐conditioned air, resulting in the fast time constant, $T_{1}=0.038$ d. In contrast, at deeper positions within the dead‐end cavity, the influence of the external air flow is diminished, and the molecular diffusion and/or water molecule absorption and transmission becomes dominant, resulting in the much slower time constant, $T_{2}=0.47$ d. Although the experimental data in Figure 2(b) show a slight deviation from the double‐exponential curve after several days, the current model with two time constants adequately captures the fundamental behavior of the equilibration process. For the purpose of this study—to recognize practical time scales for the chamber response—this approximation provides sufficient quantitative evidence that the slow component dominates the long‐term stabilization.

Due to the longer and narrower path from the dead‐end cavity to the cabinet, the assembled unit (b‐ii+b‐iii) exhibits a remarkably different behavior in Figure 2(c). The rapid initial change observed in the b‐iii unit is absent; instead, the humidity decreases at an extremely slow rate from the beginning. This indicates that the 1.5‐m venting path acts as a dominant structural resistance that effectively suppresses convective air exchange. Consequently, the equilibration time scale increases by an order of magnitude.

McEwen and Taank report that several commercially available reference ionization chambers including the TN‐30013 type tested in this study equilibrate to the ambient humidity within 1 hour when the humidity difference between the cavity and the surroundings is ∼10 % RH, and within from several hours to overnight when the humidity difference is larger than ∼50 % RH.^11^ Sato et al. also report that the equilibration is completed within ∼2 hours when the humidity is changed by 10 % RH.^12^ Our result for the assembled unit (b‐ii+b‐iii), however, reveals that the equilibration time is significantly longer than those reported in these reports. Although the modification to the original chamber wall might lead to a claim that the tested unit is not identical to the commercial one, the results for the outer shell and wall unit (b‐iii) provide a definitive counterargument. Despite the Bc unit having a much shorter and wider path than the original chamber due to its direct exposure to the ambient air, it already shows a transition to a slower time constant around a 10 % RH difference as shown in Figure 2(b); even in this open configuration, reaching full equilibrium takes on the scale of a day from 10 % RH difference. Consequently, this suggests that the humidity correction factors reported in the recent studies might have been determined under conditions where the air inside the cavity had not yet reached a true equilibrium with the external environment.

It is important to discuss the influence of ionization chamber design on the humidity equilibration time. Waterproof Farmer‐type ionization chambers commonly used in radiotherapy have a long waterproof sleeve that provides an air‐exchange path to the ambient air near the connector. Consequently, the cavity air communicates with the ambient air through a long diffusion path, and the time required to reach full equilibration is expected to be on the order of months, as observed for the assembled chamber unit (b‐ii+b‐iii) in Figure 2(c). At the other extreme, windowless free‐air chambers have the sensitive air volume directly exposed to the ambient air. This configuration is similar to that of the outer shell and wall unit (b‐iii), and therefore the humidity equilibration time is expected to be on the order of days, as shown in Figure 2(a). Between these two cases, cavity ionization chambers intended for radiation protection or primary air‐kerma standards, as well as non‐waterproof radiotherapy ionization chambers, generally have either a ventilation hole opening directly into the cavity or another short air‐exchange path to the ambient air. Accordingly, their humidity equilibration time is expected to be on the order of weeks rather than days or months.

The present results obtained in measurement I also provide the following practical guidance for users of ionization chambers in radiation dosimetry, including dose‐to‐water measurements in radiotherapy, air‐kerma or exposure measurements for radiation protection, SI‐traceable measurements using primary and secondary standards, and measurements for other related applications: (1) The humidity of the air inside the cavity is unlikely to be affected during routine dose determination if the exposure of the ionization chamber to a different environment is limited to several hours. (2) The primary factor determining the state of the air in the cavity is the long‐term storage condition of the chamber. If the storage environment is changed—for example, from an extremely low‐humidity desiccator to one at a normal humidity level, or vice versa—the cavity air will be replaced over a long period, resulting in a response change as demonstrated in measurement II.

### 4.2 Humidity correction measurement II

Although the recent studies by McEwen and Taank,^11^ and Sato et al.,^12^ reported that no apparent changes were observed down to 5.5 % RH and 10 % RH, respectively, our results show that the change is large enough to observe even with their reported instrumental uncertainties. This suggests that the time allowed to equilibrate the air inside of the cavity that they decided was too short, which is also supported by the results in measurement I. The overall trend and magnitude of our results are in closer agreement with the recommended consensus in the ICRU Report 31^9^ and the foundational studies supporting it rather than with those in the recent reports. This foundational consensus is primarily represented by three sources: the Niatel curve (a theoretical model^5^ based on the humidity‐dependent W‐values for X‐rays below 50 keV^4^) adopted in the ICRU Report 31, and the pioneering experimental results by Hofmeester et al.^8^ and Guiho et al.^6^ using graphite and brass (an alloy of copper and zinc) cavity walls, respectively. Nevertheless, a closer examination reveals two distinct differences between our observations and these three sources. First, in the high‐humidity region from 30 % RH to 100 % RH, while the Niatel and Hofmeester curves gradually decrease by 0.12%−0.14 % and the Guiho curve by ∼0.04 %, our result shows that the $k_{h}$ remains nearly constant within the uncertainties above 40 % RH, as shown in Figure 6(a). Second, in the low‐humidity region from 0 % RH up to 20 % RH, while the Niatel, Hofmeester and Guiho curves exhibit a sharp increase, our results show that the $k_{h}$ gradually increases from 0 % RH up to 40 % RH (or can be estimated up to 30 % RH) roughly linearly.

Although it is difficult to identify the decisive causes for the discrepancies between the pioneering studies and our results due to a lack of detailed information on their experiments, and due to the fact that the measured quantity is the final output of complex processes, some causes can be discussed as follows.

First, as Hofmeester and Somerwil^8^ suggested that the discrepancies between their results and the Guiho curve might arise from the wall materials, the choice of the wall materials remains a potential factor because the response to Co‐60 γ‐rays depends on the material. However, our Monte Carlo simulations presented in Figure 3(b) show that the normalized electron spectra inside the cavity are in good agreement among aluminum, graphite, and yellow brass, demonstrating that the secondary electron spectrum is independent of the wall materials. Therefore, the discrepancy between these curves can not be explained by the response to the Co‐60 γ‐rays.

Second, adsorption and desorption of water molecules on the cavity wall surface could lead to the discrepancies. Since graphite is more or less a porous material whether it is the high‐density graphite or not, the actual surface area of the wall is larger than it appears, leading to the adsorption and desorption of water molecules according to the RH of the air inside the cavity. In contrast, aluminum and brass are non‐porous, meaning that these processes are confined to the outermost surface and their contributions are expected to be smaller than those of graphite. In our result, current measurements were performed every 5 minutes, and the vacuum‐assisted gas exchange process was performed every 10 measurements; the measured currents showed no remarkable changes as seen in Figure 6(a) and Figure 4(e), indicating that adsorption and desorption from our aluminum wall are negligible. It should be noted, however, that while the Guiho curve for the brass wall is similar to ours in the middle to high humidity range, it exhibits a distinct discrepancy in the low‐humidity range.

Consequently, the discrepancies in the $k_{h}$ observed among Niatel, Hofmeester, Guiho and our measurements remain unresolved even after accounting for the response to the Co‐60 γ‐rays and the adsorption‐desorption effects. This fundamental inconsistency may necessitate a further accumulation of experimental data using high‐precision modern instrumentation, which will form a more reliable basis for the realization of the SI‐traceable air‐kerma standards.

It is interesting to note that our relative W‐value exhibited roughly linear dependence on RH. The W‐curve reported by Niatel, shown in Figure 7, was primarily measured using a free‐air chamber for X‐rays with energies below 50 keV. These experimental data, combined with theoretical considerations, eventually formed the basis for the current ICRU31 curve. The W‐curve by Niatel shows an apparent bent around 10 % RH, and the steeper slope from 0 % RH is attributed to the Jesse effect.^24^ Barnard et al. show a linear dependence of the W‐value on RH, as shown in Figure 7, by assuming that the humid air is simply a mixture of dry air and water vapor.^23^ Although the slope is steeper in our calculated results, the overall trend is consistent with the linear behavior reported by Barnard et al. rather than the curve reported by Niatel. The difference can be attributed to the electron stopping power in air. According to the NIST ESTAR database,^19^ the collision stopping power increases by more than an order of magnitude as the electron kinetic energy decreases from several hundred keV to below 50 keV. At the lower energies used by Niatel, this high linear energy transfer (LET) results in a dense, “volumetric”, energy deposition within a localized area, where collective molecular interactions and/or recombination processes may come into play. In contrast, for the high‐energy secondary electrons from Co‐60 in Figure 3(b), the relatively low stopping power leads to a sparse and linear distribution of ion pairs. In such an environment, each water molecule likely acts as an independent target, which supports the linear mixture rule and explains the consistent linear trend observed in our results.

Finally, we discuss the plausible $k_{h}$ derived in this study using the modern instrumentation. Although the lowest RH examined in our experiments did not reach 0 % RH, the measured $k_{h}$ at approximately 2 % RH was already below the internationally accepted value of 0.9970. Our fitting analysis in Figure 6(b) reveals that the extrapolated $k_{h}$ at 0 % RH ranges from 0.9962 to 0.9970, depending on the choice of fitting models and data ranges. Although this difference appears slight and may not affect most routine dosimetric works, it is critical for the primary standards that maintain extremely low uncertainties such as $\delta k_{h}=0.03$ % and $\delta K_{a}=0.13$ % as shown in Table 1. In these cases, inaccurate humidity corrections consistently shift SI‐traceable fundamental quantities from the true value. As long as the discrepancies among the pioneering works and our results (as well as those among the pioneering works themselves) remain unresolved, it is difficult to justify such low uncertainties. It should be emphasized lastly that the internationally accepted $k_{h}$ for the Co‐60 beam quality is primarily based on the two different types of studies: Niatel measured W‐value for low‐energy photons below 50 keV, and applied it to the theoretical calculation for the Co‐60 beam quality. In contrast, Hofmeester et al. and Guiho et al. appear to have assumed their “0 % RH dry air” by dehydrating air to the limit of their apparatus, lacking direct measurements of humidity of the resultant dry air, as far as can be confirmed from the literature available.

## 5 CONCLUSIONS

The plausible humidity correction factor, $k_{h}$, was re‐evaluated for the first time in about half a century with modern instrumentation. The results were more or less similar to, and at the same time, different from the pioneering works. Specifically, our results showed that $k_{h}$ increased with a more gentle slope up to 40 % RH (or possibly 30 % RH), and the intercept at 0 % RH was a slightly smaller value of 0.9962–0.9970. The relative W‐value evaluated with the measured $k_{h}$ and simulated energy deposition showed nearly linear dependence as a function of RH, which was different from the result by Niatel but similar to that of Barnard et al.

The reasons for the discrepancies between the pioneering works and our observations are not yet fully understood. To resolve this inconsistency, further independent verifications or different experimental approaches are required. Such efforts are crucial to maintaining the reliability of the current SI‐traceable units system in dosimetry.

## FUNDING INFORMATION

The authors have no funding to report.

## CONFLICT OF INTEREST STATEMENT

The authors have no relevant conflicts of interest to disclose.

## Data Availability

Research data are not shared.

## APPENDIX A

Throughout the study, the Wagner and Pruss formula^16^ was used to evaluate mass ratio between dry air and water vapor in air with RH at h [%]. The saturated water vapor pressure $p_{\mathrm{sv}}$ [Pa] at temperature T [K] is expressed as:

$$\ln\left(\frac{p_{\mathrm{sv}}}{p_{c}}\right)=c_{1}\theta +c_{2}\theta^{1.5}+c_{3}\theta^{3}+c_{4}\theta^{3.5}+c_{5}\theta^{4}+c_{6}\theta^{7.5},$$

where the coefficients are given by:

$$\begin{array}{cc} c_{1}=-7.85951783, & c_{2}=1.84408259,\\ c_{3}=-11.7866497, & c_{4}=22.6807411,\\ c_{5}=-15.9618719, & c_{6}=1.80122502. \end{array}$$

Here, $p_{c}=2.2064\times 10^{7}$ Pa is the critical pressure, $T_{c}=647.096$ K is the critical temperature, and $\theta =1-T/T_{c}$. At a total atmospheric pressure of $P_{0}=101325$ Pa, the partial pressures of water vapor ($p_{v}$) and dry air ($p_{\mathrm{dry}}$) are determined by:

$$p_{v}=\frac{hp_{\mathrm{sv}}}{100}\;\text{and}\;p_{\mathrm{dry}}=P_{0}-p_{v}.$$

## References

[1] Büermann L , Burns DT . Air‐kerma cavity standards. Metrologia. 2009;46:S24.

[2] Rogers DWO , Ross CK . The role of humidity and other correction factors in the AAPM TG‐21 dosimetry protocol. Med Phys. 1988;15:40. 10.1118/1.596292 3352550

[3] All the documents are traceable from links at https://www.bipm.org/kcdb/comparison?id=58

[4] Niatel MT . Étude expérimentale de la vapeur d'eau sur l'ionisation produite dans l'air. C R Acad Sci Paris. 1969;268:1650.

[5] Niatel MT . Influence de la vapeur d'eau sur l'ionisation de l'air dans le cas d'une chambre á cavité. C R Acad Sci Paris. 1975;281:361.

[6] Guiho JP , Pavlicsek I , Ostrowsky A , Goenvec H . Influence de l'état hygrométrique de l'air sur l'ionisation produite par les rayonnements x ou γ . C R Acad Sci Paris. 1974;278:69.

[7] Referowski Z . Influence of air humidity on ionization in a free‐air chamber. Document CCEMRI. 1977.

[8] Hofmeester GH , Somerwil A. Interim report of the influence of humidity on ionization in air. Document CCEMRI. 1977.

[9] International Commission on Radiation Units and Measurements Report No. 31. Average Energy Required To Produce An Ion Pair. 1979.

[10] Poirier AP , Douysset G . Influence of ambient humidity on the current delivered by air‐vented ionization chambers revisited. Phys Med Biol. 2006;51:4995. 10.1088/0031-9155/51/19/017 16985283

[11] McEwen MR , Taank J . Examining the influence of humidity on reference ionization chamber performance. Med Phys. 2017;44:694. 10.1002/mp.12057 28133754

[12] Sato Y , Shimizu M , Morishita Y , Sato M , Hoshina M . Experimental study of humidity effect on charge measurement of reference ionization chambers in clinical high‐energy photon beams. Med Phys. 2019;46:4177. 10.1002/mp.13665 31206700

[13] Morishita Y , Shimizu M , Takase N , Yamashita W , Sakata S . The impact of storage humidity on the response of reference‐class ionization chambers. Med Phys. 2022;49:2725. 10.1002/mp.15497 35092311

[14] Kawrakow I , Rogers DWO , Mainegra‐Hing E , et al. EGSnrc toolkit for Monte Carlo simulation of ionizing radiation transport. 2000.

[15] Kurosawa T , Takata N , Koyama Y , Kato M . AIST monograph of metrology , No. 7 2005.

[16] Wagner W and Pruss A . International Equations for the Saturation Properties of Ordinary Water Substance. Revised According to the International Temperature Scale of 1990. Addendum to J Phys Chem. Ref. Data 16, 893 (1987). J Phys Chem Ref Data. 1993;22:783.

[17] Standard Atmosphere, ISO 2533. 1975.

[18] Prohaska T , Irrgeher J , Benefield J . et al. Standard atomic weights of the elements 2021 (IUPAC Technical Report). Pure Appl Chem. 2022;94(5):573.

[19] Berger MJ . ESTAR: Computer Programs for Calculating Stopping‐Power and Range Tables for Electrons (Version 1.2). NISTIR 4999, National Institute of Standards and Technology, Gaithersburg, MD (1999), Available: https://physics.nist.gov/PhysRefData/Star/Text/download.html

[20] Bé M‐M , Chisté V , Dulieu C , et al. Table of radionuclides. Monographie BIPM‐5. 2006.

[21] Berger MJ , Hubbell JH . XCOM: Photon Cross Sections on a Personal Computer, NBSIR 87‐3597, National Bureau of Standards, Geithersburg, MD (1987), Available: https://physics.nist.gov/PhysRefData/Xcom/Text/download.html

[22] Bradbury J , Frostig R , Hawkins P , et al. JAX: composable transformations of Python+NumPy programs, http://github.com/google/jax, version 0.3.13, 2018.

[23] Barnard GP , Aston GH , Marsch ARS . National Physical Laboratory, Effects of Variations in the Ambient Air on the Calibration and Use of Ionization Dosemeters. Her Majesty's Stationery Office, London. 1960.

[24] Jesse WP , Sadauskis J . Ionization by Alpha Particles in Mixtures of Gases. Phys Rev. 1955;100:1755.

